# Supplementary figures and images for: Jorunnamycin A Suppresses Stem-Like Phenotypes and Sensitizes Cisplatin-Induced Apoptosis in Cancer Stem-Like Cell-Enriched Spheroids of Human Lung Cancer Cells
Source: Mar Drugs. 2021 May 3;19(5):261. doi: 10.3390/md19050261 (PMC8147647; doi:10.3390/md19050261)

A)

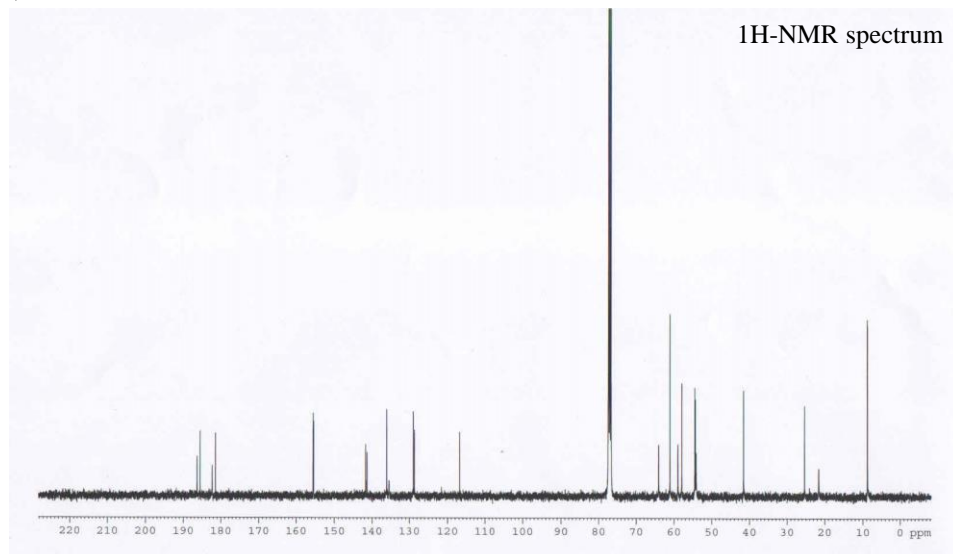

B)

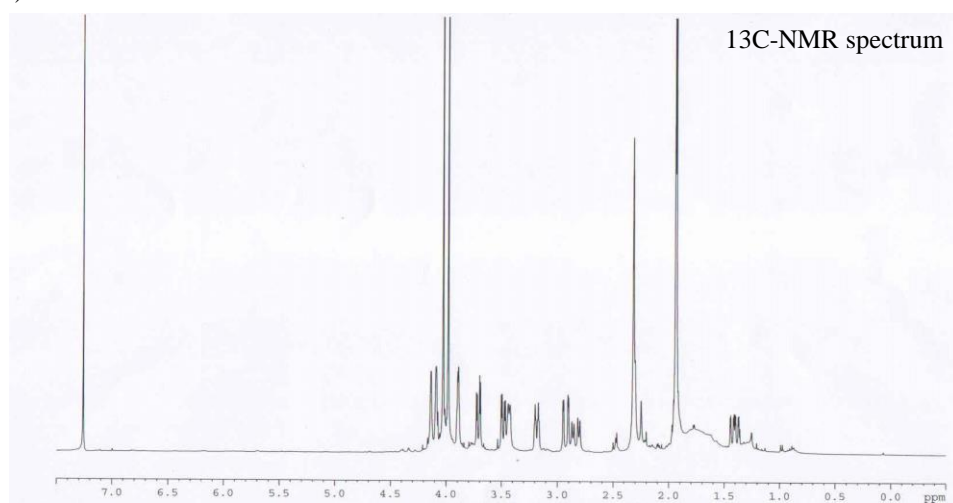

Figure S1. NMR spectrum of jorunnamycin A.

Supplement: Supplementary file 1 [file marinedrugs-19-00261-s001.zip › marinedrugs-1191288-supplementary.pdf]
